# Application of Deep Learning in Automated Analysis of Molecular Images in Cancer: A Survey

**DOI:** 10.1155/2017/9512370

**Published:** 2017-10-15

**Authors:** Yong Xue, Shihui Chen, Jing Qin, Yong Liu, Bingsheng Huang, Hanwei Chen

**Affiliations:** ^1^Guangzhou Panyu Central Hospital, Guangzhou, China; ^2^Medical Imaging Institute of Panyu, Guangzhou, China; ^3^National-Regional Key Technology Engineering Laboratory for Medical Ultrasound, Guangdong Key Laboratory for Biomedical Measurements and Ultrasound Imaging, School of Biomedical Engineering, Health Science Centre, Shenzhen University, Shenzhen, China; ^4^School of Nursing, The Hong Kong Polytechnic University, Hung Hom, Hong Kong; ^5^Intensive Care Unit, Southern Medical University Shenzhen Hospital, Shenzhen, China

## Abstract

Molecular imaging enables the visualization and quantitative analysis of the alterations of biological procedures at molecular and/or cellular level, which is of great significance for early detection of cancer. In recent years, deep leaning has been widely used in medical imaging analysis, as it overcomes the limitations of visual assessment and traditional machine learning techniques by extracting hierarchical features with powerful representation capability. Research on cancer molecular images using deep learning techniques is also increasing dynamically. Hence, in this paper, we review the applications of deep learning in molecular imaging in terms of tumor lesion segmentation, tumor classification, and survival prediction. We also outline some future directions in which researchers may develop more powerful deep learning models for better performance in the applications in cancer molecular imaging.

## 1. Introduction

With increasing incidence and mortality, cancer has always been a leading cause of death for many years. According to American Cancer Society, there are around 1,685,210 new cases and 595,690 deaths in 2016 [1]. It was reported that the 5-year survival rate for the cancer patients diagnosed in early stage was as high as 90% [2]. In this regard, early and precise diagnosis is critical for better prognosis of cancer.

Molecular imaging is an imaging technique to visualize, characterize, and measure biological procedures at molecular and/or cellular level [3] and has been considered as a powerful tool for early detection of cancer. Compared with anatomical imaging techniques, molecular imaging is more promising in diagnosing cancer in the early stage, as it is capable of signaling the molecular or physiological alterations in cancer patients which may happen before the obvious anatomical changes. Molecular imaging is also helpful in individualized therapy as it can reflect the treatment response at the molecular level. Therefore, molecular imaging has been widely used in cancer management.

The current molecular imaging modalities in clinical practice include contrast-enhanced computed tomography (CT), contrast-enhanced magnetic resonance (MR) imaging, MR spectroscopy, and nuclear medicine such as single photon emission computed tomography (SPECT) and positron emission tomography (PET). Visual assessment conducted by the radiologists is the most common way to analyze these images. However, subtle changes in molecular images may be difficult to detect by visual inspection as the target-to-background ratio in these images is not that significant. In addition, visual interpretation by clinicians not only is time-consuming but also usually causes large variations across interpreters due to the different experience.

The emerging intelligent techniques are of great potential in solving these problems by making the image interpretation automated. Machine learning-based image processing has been widely used in the domain of medical imaging analysis. Conventional machine learning techniques require the artificial intervention of feature extraction and selection and thus are still somehow subjective. In addition, the subtle and distributed changes may be ignored with artificial feature calculation and selection. Fully automated techniques are expected to integrate the local and global information for more accurate interpretation. Deep learning as a state-of-the-art machine learning technique may solve the challenges aforementioned by abstracting higher level features and improving the predictions from data with deep and complex neural network structures [4].

### 1.1. Deep Learning

The deep architectures and algorithms have been summarized [5, 6]. Compared with the conventional machine learning techniques, deep learning has shown some advantages [5, 6]. First, deep learning can automatically acquire much richer information in a data-driven manner and these features are usually more discriminative than the traditional hand-crafted features. Second, deep learning models are usually trained in an end-to-end way; thus the feature extraction, feature selection, and classification can be conducted and gradually improved through supervised learning in an interactive manner [7]. Therefore, deep learning is promising in a wide variety of applications including cancer detection and prediction based on molecular imaging, such as in brain tumor segmentation [8], tumor classification, and survival prediction. Deep learning-based automated analysis tools can greatly alleviate the heavy workload of radiologists and physicians caused by the popularity of molecular imaging in early diagnosis of cancer as well as enhance the diagnostic accuracy, especially when there exist subtle pathological changes that cannot be detected by visual assessment.

Deep learning-based methods mainly include convolutional neural networks (CNN), restricted Boltzmann machines (RBMs), autoencoder, and sparse coding [9]. Among them, CNN and autoencoder have been widely applied in cancer molecular imaging. To our best knowledge, CNN models are especially the most commonly used methods with more powerful architecture and flexible configuration to learn more discriminative features for more accurate detection [10]. A typical CNN architecture for image processing consists of three types of neural layers, including the convolutional layers, the pooling layers, and the fully connected layers. The convolutional layer contains a series of convolution filters, which can learn the features from training data through various kernels and generate various feature maps. A pooling layer is generally applied to reduce the dimension of feature maps and network parameters, and a fully connected layer is used to combine the feature maps as a feature vector for classification. Because the fully connected layers require a large computational effort during the training process, they are often replaced with convolutional layers to accelerate the training procedure [11, 12]. On the other hand, autoencoder is based on the reconstruction of its own inputs and is optimized by minimizing the reconstruction error [9].

### 1.2. Literature Selection and Classification

The papers on diverse applications of deep learning in different molecular imaging of cancer published from 2014 onwards were included. This review contains 25 papers and is organized according to the application of deep learning in cancer molecular imaging, including tumor lesion segmentation, cancer classification, and prediction of patient survival.  Table 1 summarizes the 13 different studies on tumor lesion segmentation, while Table 2 summarizes the 10 different studies on cancer classification. Two interesting papers on prediction of patient survival are also reviewed (Table 3). To our best knowledge, there is no previous work making such a comprehensive review on this issue. In this regard, we believe this survey can present radiologists and physicians with the application status of advanced artificial intelligent techniques in molecular images analysis and hence inspire more applications in clinical practice. Biomedical engineering researchers may also benefit from this survey by acquiring the state of the art in this field or inspiration for better models/methods in future research.

## 2. Deep Learning in Tumor Lesion Segmentation

Accurate tumor segmentation plays an essential role in treatment planning and the assessment of radiotherapy treatment efficacy. Studies have focused on tumor segmentation based on deep learning and molecular imaging, aiming at providing powerful tools for clinicians to automatically and accurately delineate lesions for better diagnosis and treatment.

Postcontrast T1W-MRI is a molecular imaging technique, which is of great help in delineating the enhancing lesions and necrotic regions. Indeed, deep learning models have been trained with multimodality MRI data, including contrast-enhanced T1W, to achieve better performance in brain tumor segmentation.

Deep neural networks (DNN) were found effective for task-specific high-level feature learning [13] and thus were used to detect MRI brain-pathology-specific features by integrating information from multimodal MRI. In four brain tumor patients, Zhou et al. [14] applied the incremental manifold learning [15] and DNN models to predict tumor progression, respectively. For incremental manifold learning system, feature extraction consists of three parts: landmark selection using statistical sampling methods, manifold skeleton identification from the landmarks, and inserting out-of-bag samples into the skeleton with Locally Linear Embedding (LLE) algorithm [16, 17]. Fisher score and Gaussian mixture model (GMM) were employed for feature selection and classifier training, respectively. For DNN, feature extraction, feature selection, and classification were achieved in the same deep model by pretraining the model in an unsupervised way and then fine-tuning the model parameters with label. Though the average result produced by deep neural network models was just a little better than that of the incremental manifold learning due to the limited training samples, DNN still demonstrated great potential for the clinical applications.

Various 2D CNN and 3D CNN models were proposed for brain tumor segmentation and were evaluated on public databases such as brain tumor segmentation (BRATS) challenges [18]. The data from BRATS consists of four MRI sequences, including T1W, T1-postcontrast (T1c), T2W, and FLAIR.

2D CNNs were firstly applied for 3D brain tumor segmentation with consideration of less modification to the existing models and less computational load. Zikic et al. [19] used a standard CNN architecture with two convolutional layers: one followed by a max-pooling layer and the other followed by a fully connected layer and a softmax layer. Standard intensity preprocessing was used to remove scanner difference but without any postprocessing for the CNN output. They tested the proposed method on 20 high-grade cases from the training set of the BRATS 2013 challenge and obtained promising preliminary results. Actually, the 2D CNNs may not be sufficiently powerful for 3D segmentation; thus the information extracted axially, sagittally, and coronally should be combined. Lyksborg et al. [20] proposed a method based on an ensemble of 2D CNNs to fuse the segmentation from three orthogonal planes. The GrowCut algorithm [21] was also applied to smooth the segmentation of the complete tumor for postprocessing. They achieved better performance than axially trained 2D network and the ensemble method without GrowCut on BRATS 2014. It is worth noting that the combination of information from different orthogonal planes and the application of postprocessing algorithm contributed to this enhancement.

Instead of applying a known postprocessing algorithm such as Markov Random Fields (MRF) [22] for smoother segmentation, useful information provided by the neighboring voxels can also be integrated through the local structure prediction by taking the local dependencies of labels into consideration. Dvořák and Menze [23] proposed a method combining local structure prediction and CNN, where *K*-means was used for generation of the label patch dictionary and then CNN was used for input prediction. Both labels of the neighboring pixels and the center pixels were taken into account in this method. They obtained state-of-the-art results on the BRATS 2014 dataset for brain tumor segmentation.

**Figure 1:**
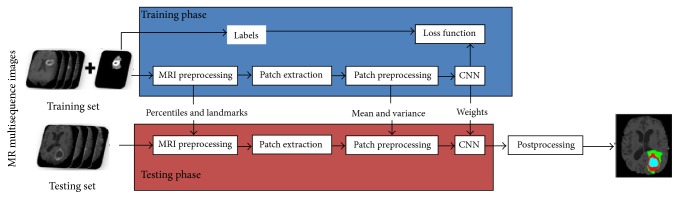
Framework of the proposed method. Image courtesy of Sérgio Pereira, Adriano Pinto, Victor Alves, and Carlos A. Silva, University of Minho.

**Figure 2:**
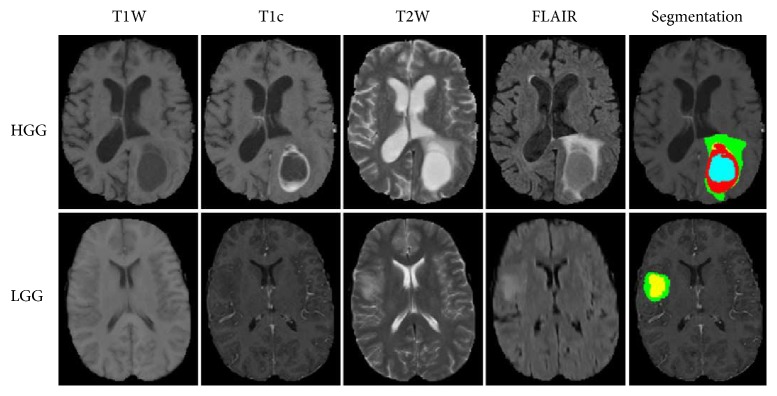
Example of brain tumor segmented into different tumor classes (green, edema; blue, necrosis; yellow, nonenhancing tumor; red, enhancing tumor) by the proposed method. Image courtesy of Sérgio Pereira, Adriano Pinto, Victor Alves, and Carlos A. Silva, University of Minho.

The main challenges of CNN lie in overfitting caused by the large amount of parameters and time-consuming training process. Some studies have applied appropriate training strategies to solve these problems. Pereira et al. [24, 25] used a deep CNN for the segmentation of gliomas in multisequence MRI and applied Dropout [26], leaky rectifier linear units, and small convolutional kernels to address overfitting (Figure 1). They used different CNN architectures for low-grade glioma (LGG) and high-grade glioma (HGG). The convolutional layers were halved in the architecture for LGG. Data augmentation was employed in this study and was found useful. The examples of segmentation were shown in Figure 2. They obtained the first place on a 2013 public challenge dataset, and the second place in an on-site 2015 challenge. Proper structure improvement can accelerate the training process. Havaei et al. [27] proposed a variety of CNN models based on two-pathway and cascaded architectures, respectively, for tackling brain tumor segmentation, incorporating both local features and more global contextual features simultaneously. Their CNN allowed a 40-fold speed up using a convolutional implementation of a fully connected layer as a final layer. In addition, a 2-phase training procedure can solve the problem due to the imbalance of tumor labels. Compared to the currently published state-of-the-art methods, the results that Havaei et al. [27] reported on the 2013 BRATS test dataset was over 30 times faster. In addition, the cascaded method made refinement for the probability maps generated by the base model, which made them one of the top 4 teams in BRATS 2015 [28].

To make full use of 3D information, 3D CNNs have been also developed in the recent two years for better segmentation performance. With consideration of the limitations in the existing models, a 3D CNN with a dual pathway and 11 layers was devised by Kamnitsas et al. [29]. The computational load of processing multimodal 3D data was also reduced by an efficient training scheme with dense training [30]. Due to conditional random fields (CRF) with the strong regularization ability for improving the segmentation, a 3D fully connected CRF [31] was incorporated with the proposed multiscale 3D CNN to remove false positive effectively. The proposed model was employed on BRATS 2015 for generalization testing and achieved top ranking performance.

Limited sample size is a key factor affecting the CNN performance. Yi et al. [32] proposed a 3D fully CNN with a modified nontrained convolutional layer that was able to achieve the enlargement of the training data size by incorporating information at pixel level instead of patient level. The proposed method was evaluated on BRATS 2013 and BRATS 2015 and achieved superior performance.

Casamitjana et al. [33] tested three different 3D fully connected CNNs on the training datasets of BRATS 2015. The three models were based on the VGG architecture [34], learning deconvolution network [35], and a modification of multiscale 3D CNN [29] presented above, respectively. All these models obtained promising preliminary results, with an accuracy of 99.69%, 99.71%, and 99.71%, respectively.

Zhao et al. [36] proposed a method based on the integration of fully CNN and CRF [37]. In addition, this slice-by-slice tumor segmentation method enabled the acceleration of the segmentation process. The proposed method finally achieved comparative performance with the combination of FLAIR, T1c, and T2 images from BRATS 2013 and BRATS 2016 than those results on the combination of FLAIR, T1c, T1, and T2 images, which suggested that the proposed method was powerful and promising.

The requirement of large training database with manual labels constrains the application of CNN-based models, since manual annotations are usually unavailable or intractable in a large dataset. Therefore, semisupervised or weakly supervised learning should be considered as a substitute to supervised learning. Autoencoder-based models have shown advantage in model training with unlabeled data. Alex et al. [38] proposed a method based on weakly supervised stacked denoising autoencoders to segment brain lesion as well as reduce false positive. Due to the LGG samples in a limited size, transfer learning was employed in this study. LGG segmentation was achieved using a network pretrained by large HGG data and fine-tuned by limited data from 20 LGG patients. The proposed method achieved competitive performance on unseen BRATS 2013 and BRATS 2015 test data.

Besides lesion detection, accurate segmentation is also essential to radiotherapy planning. For head and neck cancer, Ibragimov et al. [39] proposed a classification scheme for automated segmentation of organs-at-risk (OARs) and tongue muscles based on deep learning and multimodal medical images including CT, MR, and PET. The promising results presented in this comprehensive study suggested that deep learning has great potential in radiotherapy treatment planning.

Regarding tumor segmentation, deep learning models can learn more abstract information or high-level feature representation from images and thus achieve better performance than those methods based on shallow structures. In addition, the combination of deep and shallow structures is more powerful than the single deep learning model. However, the challenges of deep learning models mainly lie in how to avoid overfitting and to accelerate the training process. Specific techniques such as Dropout, Leaky Rectifier Linear Units, and small convolutional kernels have been developed to address overfitting, and proper improvements of deep learning architectures have been made to accelerate the training. It is worth noting that the dataset used in these studies were multimodal; thus the information provided by the molecular imaging and anatomical imaging can be integrated effectively. The integrated information may be utilized efficiently by deep learning models and thus contribute to better segmentation performance.

Since 2013, the dataset of BRATS benchmark was divided into five classes according to the pathological features presented in different modalities. Each class has a specific manual label, including healthy, necrosis, edema, and nonenhancing and enhanced tumor. In addition, three tumor regions were defined as the gold standard of segmentation, including complete tumor region (necrosis, edema, and nonenhancing and enhanced tumor), core tumor region (necrosis and nonenhancing and enhanced tumor), and enhancing tumor region (enhanced tumor). Generally, deep learning models achieved best performance in HGG segmentation. The relatively poor performance in LGG segmentation may be caused by sample imbalance, since less LGG patients were included in the BRATS benchmark. Besides, the inherent class imbalance of the dataset was also likely to lead to the poor performance in enhancing tumor region segmentation. For example, the real proportion of five classes in BRATS 2015 is 92.42%, 0.43%, 4.87%, 1.02%, and 1.27% for healthy, necrosis, edema, and nonenhancing and enhancing tumor, respectively [29].

## 3. Deep Learning in Cancer Classification

**Figure 3:**
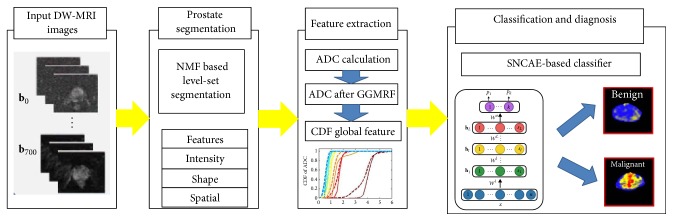
Framework of the DW-MRI CAD system for prostate cancer classification. Image courtesy of Islam Reda et al.

For early detection of prostate cancer, deep learning techniques such as CNN and stacked autoencoders (SAE) have been applied on diffusion-weighted magnetic resonance images (DW-MRI) and multiparametric MRI. Reda et al. [40] used the cumulative distribution function (CDF) of refined apparent diffusion coefficient (ADC) for the prostate tissues at different *b*-values as global features and trained a deep autoencoder network with stacked nonnegativity-constrained autoencoders (SNCAE) for classification of benign and malignant prostate tumors. Reda et al. [41] also proposed an automated noninvasive CAD system based on DW-MRI and SNCAE for diagnosing prostate cancer. There were three steps for the proposed scheme: (i) localizing and segmenting prostate with a deformable nonnegative matrix factorization- (NMF-) based model; (ii) constructing the CDF of estimated ADC as extracted discriminatory characteristics; (iii) classifying benign and malignant prostates with SNCAE classifier (Figure 3). The SNCAE-based method proposed by Reda et al. [41] has achieved excellent classification performance on the DW-MRI data from 53 subjects, but this method still needs several preprocessing steps leveraging hand-crafted features, which may greatly affect the computational load of the classification. Zhu et al. [42] proposed a method based on SAE and multiple random forest classifiers for prostate cancer detection, in which a SAE-based model was employed to extract latent high-level feature representation from multiparametric MR images for the first step; then multiple random forest classifiers were implemented for refinement of prostate cancer detection results. Though the proposed method has been proved effective on 21 prostate cancer patients, it should still be further validated on a large sample.

CNN has been widely used in brain tumor evaluation, grading, and detection. The codeletion of chromosome arms 1p/19q status prediction is clinically important for it plays an important role in treatment planning of LGG. To find out a potential noninvasive alternative to surgical biopsy and histopathological analysis, Akkus et al. [43] applied multiscale CNN to predict 1p/19q status for effective treatment planning of LGG based on T1c and T2W images. The results suggested that artificial data augmentation potentially enhance the performance by improving generalization ability of the multiscale CNN and avoiding overfitting. Pan et al. [44] compared the performance of Neural Networks (NN) and CNN for brain tumor grading. They found that CNN outperformed NN for grading, but the more complex structure of CNN did not show better results in the experiment. Because different treatment strategies are needed for glioblastoma and primary central nervous system lymphoma, it is clinically important to differentiate them form each other. Hirata et al. [45] applied CNN for differentiation of brain FDG PET between glioblastoma and primary central nervous system lymphoma (PCNSL). The method supplemented by the manual-drawing ROIs achieved higher overall accuracy on both slice-based and patient-based analysis than that without ROI masking, which suggested that CNN may be more powerful combined with an appropriate tumor segmentation technique. To achieve fully automated quantitative analysis of the brain tumor metabolism based on ^11^C-methionine (MET) PET, Hirata et al. [46] applied CNN to extract the tumor slices from the whole brain images based on MET PET and achieved better classification performance than maximum standardized uptake value (SUV_max_) based method. With high specificity, the CNN technique has been proven to be effective in detecting the slices with tumor lesions on MET PET from 45 glioma patients as a slice classifier.

CNN has been applied in computer-aided detection of lung tumors. Teramoto et al. [47] proposed an ensemble false positive- (FP-) reduction method based on conventional shape/metabolic features and CNN technique. The proposed method removed approximately half of the FPs in the previous methods. Wang et al. [48] compared CNN and four classical machine learning methods (random forests, support vector machines, adaptive boosting, and artificial neural network) for the classification of mediastinal lymph node metastasis of non-small-cell lung cancer (NSCLC) from ^18^F-FDG PET/CT images. In this study, it was reported that CNN was not significantly different from the best traditional methods or human doctors for classifying mediastinal lymph node metastasis of NSCLC from PET/CT images.

The training data of a small size is considered as the main reason for the limited performance of deep learning. Khan and Yong [49] reported that the hand-crafted features outperformed the deep learned features in medical image modality classification with small datasets. Cho et al. [50] presented a study on determining how much training dataset is necessary to achieve high classification accuracy. With CNN, the accuracy of different body part (like brain, neck, shoulder, etc.) classification based on CT images was greatly improved as the training sample size increased from 5 to 200. CNN with deeper architecture might outperform other approaches by increasing the training data and applying the training strategy of transfer learning and fine-tuning [13]. Transfer learning has been used in medical imaging applications, as a key strategy to solving the problem of insufficient training data. Antropova et al. [51] used the CNN architecture ConvNet pretrained by AlexNet on the ImageNet database for breast cancer classification on dynamic contrast-enhanced MR images (DCE-MRI) and showed that transfer learning can enhance the predicting performance of breast cancer malignancy. Transfer learning is commonly used in CNN-based models for network initialization when the training data is limited and the fine-tuning of the parameters is usually required for the specific tasks. However, the theoretical understanding on why transfer learning accelerates the learning process and improves the generalization ability remains unknown.

Deep learning has been applied for the classification of prostate cancer, brain tumor, lung tumor, and breast cancer based on molecular imaging. Most studies mentioned above have proven the better performance of deep learning, but a few studies indicated that the results achieved by deep learning models were not significantly better than the best conventional methods. The various results suggested that deep learning models with well-designed architecture have great potential to achieve excellent classification performance. Besides, deep learning models may achieve better performance combined with shallow structures for contextual information integration. Sufficient training data is required to prevent overfitting and to improve generalization ability, which is still a challenge in many applications. In practice, data augmentation, pretraining, and fine-tuning were often applied to tackle these problems.

## 4. Deep Learning in Survival Prediction

Besides tumor segmentation and classification, deep learning has also been employed in predicting patients' survival. Liu et al. [52] applied the CNN-F architecture [53] pretrained on ILSVRC-2012 with the ImageNet dataset for predicting survival time based on brain MR images, achieving the highest accuracy of 95.45%. Paul et al. [54] predicted short- and long-term survivors of non-small-cell adenocarcinoma lung cancer on contrast CT images, with 5 postrectified linear unit features extracted from a VGG-F pretrained CNN and 5 traditional features. They obtained an accuracy of 90% and AUC of 0.935. With high accuracy, pretrained CNN architectures may have potential to predict survival of cancer patients in the future.

## 5. Trends and Challenges

Along with the promising performance achieved by deep learning in molecular imaging of cancer, challenges and inherent trends have been posed in the following aspects.

Firstly, although deep learning has outperformed other methods based on shallow structures and achieved promising results, the underlying theory needs to be further investigated. The numbers of layers and nodes in each layer are usually determined by experience, and the learning rate and the regularization strength are chosen subjectively. Two key components should be considered for devising the deep learning model: the architecture and the depth. For model configuration, different architectures should be evaluated for the specific task.

Secondly, the insufficient data is a common challenging when employing deep learning techniques in many applications. In this case, effective training schemes should be exploited to cope with this problem. The strategies of data augmentation, pretraining, and fine-tuning have been applied in some studies, but the underlying mechanism of some of these strategies still remains unclear. It is suggested that public database of molecular imaging should be established. In addition, integrating information from multimodal imaging may improve the model performance. Moreover, it is worth noting that the sample imbalance should be avoided during training process by keeping the balance of sample size between the subtypes of a specific cancer.

Thirdly, as manual annotations are difficult or expensive in a large dataset, semisupervised and unsupervised learning are highly required in the future development [4]. Unsupervised learning and the generation of features layer by layer has made the deep architecture training possible and has improved the signal-to-noise ratio at lower levels compared to supervised learning algorithm [55–57], while semisupervised methods may achieve a good generalization capability and superior performance compared to unsupervised learning [14].

Finally, given that the abstract information extracted by deep learning models is not well understood, the correlation between the high-level feature and clinical characteristics in molecular imaging should be established to increase the reliability of deep learning techniques. Typically, these clinical characteristics of molecular imaging include the expression and activity of specific molecules (e.g., proteases and protein kinases) and biological processes (e.g., apoptosis, angiogenesis, and metastasis) [58]. Ideally, the relationship between the features output in each layer and the clinical characteristics acquired by surgical biopsy and pathological analysis is expected to be validated. In that case, the layers without significant correlation with clinical characteristics can be removed, which may increase the effectiveness of the proposed model and reduces computational resources.

## 6. Conclusion

We present a comprehensive review of diverse applications of deep learning in molecular imaging of cancer. The applications of deep learning in cancer mainly included tumor lesion segmentation, cancer classification, and survival prediction. CNN-based models are most commonly used in these studies and have achieved promising results. Despite the encouraging performance, studies are still required for further investigations about model optimization, public database establishment, and unsupervised learning as well as of the correlation between high-level features and clinical characteristics of cancer. In order to solve these problems, clinicians and engineers should work together by taking complementary expertise and advantages. In conclusion, deep learning as a promising and powerful tool will aid and improve the application of molecular imaging in cancer diagnosis and treatment.

## Figures and Tables

**Table 1:** Comparison of the performance of different deep learning-based segmentation methods.

Publication	Type of images	Proposed methods		Comparison baseline	
		Method	Results	Method	Results
Zhou et al. [14]	Multiple MRI	DNN	average = 0.864 (average of SEN, SPE and PRE)	Manifold learning	Average = 0.849

Zikic et al. [19]	BRAST 2013	CNN	HGG (complete): ACC = 0.837 ± 0.094	RF	HGG: ACC = 0.763 ± 0.124

Lyksborg et al. [20]	Multimodal MRI	CNN	Dice = 0.810, PPV = 0.833, SEN = 0.825	Axially trained 2D network	Dice = 0.744, PPV = 0.732, SEN = 0.811

Dvořák and Menze [23]	BRATS 2014	CNN	HGG (complete): Dice = 0.83 ± 0.13	—	—

Pereira et al. [24]	BRATS 2015	CNN	LGG (complete): DSC = 0.86, PPV = 0.86, SEN = 0.88 HGG (complete): DSC = 0.87, PPV = 0.89, SEN = 0.86 Combined: DSC = 0.87, PPV = 0.89, SEN = 0.86	—	—

Pereira et al. [25]	BRATS 2013	CNN	DSC = 0.88, PPV = 0.88, SEN = 0.89	Tumor growth model + tumor shape prior + EM	DSC = 0.88, PPV = 0.92, SEN = 0.84

Havaei et al. [27]	BRAST 2013	INPUTCASCADECNN	Dice = 0.88, SPE = 0.89, SEN = 0.87	RF	Dice = 0.87, SPE = 0.85, SEN = 0.89

Kamnitsas et al. [29]	BRATS 2015	Multiscale 3D CNN + CRF	DSC = 0.849, PREC = 0.853, SEN = 0.877	—	—

Yi et al. [32]	BRATS 2015	3D fully CNN	ACC = 0.89	GLISTR algorithm	ACC = 0.88

Casamitjana et al. [33]	BRATS 2015	Three different 3D fully connected CNNs	ACC = 0.9969/0.9971/0.9971	—	—

Zhao et al. [36]	BRATS 2013	3D fully CNN + CRF	Dice = 0.87, PPV = 0.92, SEN = 0.83	CNN	Dice = 0.88, PPV = 0.88, SEN = 0.89

Alex et al. [38]	BRATS 2013/2015	SDAE	ACC = 0.85 ± 0.04/0.73 ± 0.25	—	—

Ibragimov et al. [39]	CT, MR and PET images	CNN	Dice = 0.818	—	—

*Notes*. BRAST = multimodal brain tumor segmentation dataset, including four MRI sequences (T1W, T1-postcontrast (T1c), T2W, and FLAIR); CNN = convolutional neural networks; HGG = high-grade gliomas; ACC = accuracy; RF = random forests; DNN = deep neural network; Average = the average values of sensitivity, specificity, and precision; LGG = low-grade gliomas; PPV = positive predictive value; SEN = sensitivity; DSC = dice similarity coefficient; INPUTCASCADECNN = cascaded architecture using input concatenation; EM = expectation maximization algorithm; SPE = specificity; PREC = precision; GLISRT (glioma image segmentation and registration); CRF = conditional random fields; SDAE = stacked denoising autoencoder.

**Table 2:** Comparison of the performance of deep learning-based classification methods.

Publication	Type of images	Proposed methods		Comparison baseline	
		Method	Results	Method	Results
Reda et al. [40]	DW-MRI	SNCAE	ACC = 1, SEN = 1, SPE = 1	*K* ^*∗*^	ACC = 0.943, SEN = 0.943, SPE = 0.944

Reda et al. [41]	DW-MRI	SNCAE	ACC = 1, SEN = 1, SPE = 1, AUC ≈ 1	*K* ^*∗*^	ACC = 0.943, SEN = 0.962, SPE = 0.926, AUC = 0.93

Zhu et al. [42]	T2-weighted, DWI and ADC	SAE	SBE = 0.8990 ± 0.0423, SEN = 0.9151 ± 0.0253, SPE = 0.8847 ± 0.0389	HOG features	SBE = 0.8814 ± 0.0534, SEN = 0.9191 ± 0.0296, SPE = 0.8696 ± 0.0563

Akkus et al. [43]	T1-postcontrast (T1C) and T2	Multiscale CNN	ACC = 0.877, SEN = 0.933, SPE = 0.822	—	—

Pan et al. [44]	BRATS 2014	CNN	SEN = 0.6667, SPE = 0.6667	NN	SEN = 0.5677, SPE = 0.5677

Hirata et al. [45]	FDG PET	CNN	ACC = 0.88	SUV_max_	ACC = 0.80

Hirata et al. [46]	MET PET	CNN	ACC = 0.888 ± 0.055	SUV_max_	ACC = 0.66

Teramoto et al. [47]	PET/CT	CNN	SEN = 0.901, with 4.9 FPs/case	Active contour filter	SEN = 0.901, with 9.8 FPs/case

Wang et al. [48]	FDG PET	CNN	ACC = 0.8564 ± 0.0809, SEN = 0.8353 ± 0.1385, SPE = 0.8775 ± 0.1030 AUC = 0.9086 ± 0.0865	AdaBoost + D13	ACC = 0.8505 ± 0.0897, SEN = 0.8565 ± 0.1346, SPE = 0.8445 ± 0.1261 AUC = 0.9143 ± 0.0751

Antropova et al. [51]	DCE-MRI	CNN ConvNet	AUC = 0.85	—	—

*Notes*. DW-MRI = diffusion-weighted magnetic resonance images; SNCAE = stacked nonnegativity-constrained autoencoders; ACC = accuracy; SEN = sensitivity; SPE = specificity; AUC = area under the receiver operating characteristic curve; *K*^*∗*^ = *K*-Star, a classifier implemented in Weka toolbox [59]; DWI = diffusion-weighted imaging; ADC = apparent diffusion coefficient; SAE = stacked autoencoder; SBE = section-based evaluation; HOG = histogram of oriented gradient; CNN = convolutional neural network; BRATS = multimodal brain tumor segmentation dataset, including four MRI sequences (T1W, T1-postcontrast, T2W, and FLAIR); NN = neural network; FDG = fluorodeoxyglucose; PET = positron emission tomography; SUV_max_ = maximum standardized uptake value; MET = ^11^C-methionine; CT = computed tomography; FP = false positive; AdaBoost = adaptive boosting; D13 = 13 diagnostic features.

**Table 3:** Comparison of the performance of deep learning-based survival prediction methods.

Publication	Type of images	Proposed methods		Comparison baseline	
		Method	Results	Method	Results
Liu et al. [52]	MRI	CNN + RF	ACC = 0.9545	CHF	ACC = 0.9091
Paul et al. [54]	Contrast-enhanced CT	CNN + SUFRA + RF	AUC = 0.935	TQF + DT	AUC = 0.712

*Notes*. MRI = magnetic resonance imaging; CNN = convolutional neural network; RF = random forest; ACC = accuracy; CHF = conventional histogram feature; CT = computer tomography; SUFRA = symmetric uncertainty feature ranking algorithm [60]; AUC = area under the receiver operating characteristic curve; TQF = traditional quantitative features; DT = decision tree.
